# Retinitis Pigmentosa: Disease Mechanisms, Diagnosis, and Therapies

**DOI:** 10.1155/2015/819452

**Published:** 2015-06-11

**Authors:** Xinhua Shu, Ji-jing Pang, Houbin Zhang, David Mansfield

**Affiliations:** ^1^Department of Life Sciences, Glasgow Caledonian University, Glasgow G4 0BA, UK; ^2^School of Ophthalmology & Optometry, The Eye Hospital, Wenzhou Medical University, Wenzhou, Zhejiang 325027, China; ^3^Department of Ophthalmology, College of Medicine, University of Florida, Gainesville, FL 32610, USA; ^4^Sichuan Provincial Key Laboratory for Disease Gene Study, Hospital of University of Electronic Science and Technology of China and Sichuan Provincial People's Hospital, Chengdu, Sichuan 610072, China; ^5^Department of Ophthalmology, Inverclyde Royal Hospital, Greenock PA16 0XN, UK

Retinitis pigmentosa (RP) is the most genetically and phenotypically heterogeneous disorder, characterized by the progressive death of photoreceptor cells. In recent years, huge advances have been made in understanding the disease mechanisms, identifying causal genes, and developing therapeutic strategies for this disorder. This special issue updates the knowledge of RP and presents original clinical and experimental research.

Photoreceptor cell death is known to be characterized at the early stage by caspase dependent or independent apoptosis and at late stage by necrosis, but the molecular mechanisms are not fully understood. In this special issue, S. R. Patnaik et al. review the functional role of RPGR protein complex in the pathogenesis of RP. Disease mechanisms are further elucidated in mouse (Q. Zheng et al.) and zebrafish models (Y. Liu et al. and S. Akhtar et al.). R. Migliorini et al. discuss RP associated syndromes, such as impaired ocular motility. E. Strobbe et al. report a correlation between ocular inflammation and endothelin (ET-1) plasma levels in early RP patients and suggest that anti-inflammatory therapy may slow the progression of RP.

With the development of new technologies such as next generation sequencing (NGS), more and more mutant genes that cause retinal degenerative diseases have been found. B. Gong et al. reported here a recessive RDH12 mutation identified by exome sequencing in severe early onset RP patients, which further verifies the application of NGS for molecular diagnosis of RP. Meanwhile, many naturally occurring or genetically engineered animal models have shown gene mutations and phenotypes similar to human inherited retinal diseases, which has led to the development of a variety of therapeutic strategies for those inherited diseases regarded traditionally as incurable.

Following the success of Leber congenital amaurosis 2 (LCA2) gene therapy clinical trials, more and more vector based gene therapy clinical trials have been performed on a variety of retinal conditions including RP with MERTK mutation. AAV-mediated gene replacement therapy shows great potential to treat patients in the early stage of the disease. Gene replacement therapy combined with other approaches like treatment with histone deacetylases inhibitors (reviewed by H. Zhang et al.) or antiapoptotic/inflammatory chemicals or natural products, which can extend the therapeutic window in middle to late stages of those patients, is a potentially promising strategy for improving photoreceptor function and significantly slowing the process of retinal degeneration. Cell replacement is a promising therapeutic strategy for RP. Successful cell replacement treatments have been done in RP animal models, providing hope for RP patients and their families. Chinese medicine has a long history in the treatment of RP; combinational treatment of Chinese medicine with Western medicine may further reduce the progression of RP (reviewed by J. Xu and Q. Peng).

*Xinhua Shu* *Xinhua Shu* *Ji-jing Pang* *Ji-jing Pang* *Houbin Zhang* *Houbin Zhang* *David Mansfield* *David Mansfield*

